# The Gynecic Element in Psychiatry—with Suggestions for Asylum Reform

**Published:** 1889-05

**Authors:** Charles A. L. Reed

**Affiliations:** Professor of Surgical Diseases of Women at the Cincinnati College of Medicine and Surgery and at the Cincinnati Polyclinic; Fellow of the American Association of Obstetricians and Gynecologists; 311 Elm Street


### Buffalo Medical and Surgical Journal

Vol. XXVIII. MAY, 1889. No. 10

### Original Communications.

### THE GYNECIC ELEMENT IN PSYCHIATRY—WITH SUGGESTIONS FOR ASYLUM REFORM.

By CHARLES A. L. REED, M. D.,

Professor of Surgical Diseases of Women at the Cincinnati College of Medicine and Surgery and at the Cincinnati Polyclinic; Fellow of the American Association of Obstetricians and Gynecologists.

In responding to your kind invitation to address you—an honor for which I am thankful—I feel called upon at the very outset to apologize for violating what I take to be one of the canons of good taste. In announcing the title of my discourse, I avail myself of the technical phraseology, which, I think, characterizes one of the pernicious tendencies of the medical profession to-day. It would have satisfied any of we plain-spoken gynecologists to have stated that I would talk about the relation of diseases of the female generative organs to the causation and treatment of insanity. It is one of my objects, however, to engage the attention of our friends the alienists, and to do so, I have thought it best to employ at least a little of what has become almost their vernacular. And I may state just here, that my object in inviting their attention is not to quarrel with them, for they have done something for humanity, but, through them, to attack a system of which they are the products and exponents—a system which, though better than it was, is still behind the times,* in that it fails to employ, in any adequate way, the latest and best-proved revelations of science in the treatment of the insane.

---

* Read before the Alumni Association of Niagara University, Medical Department, Buffalo, N. Y., April 9, 1889.

It is my business, on this occasion, to deal with but one count in this general indictment, viz.: that, with but few exceptions, the causal relation of diseases of the female generative organs to mental disorders in women is practically without recognition in the treatment of the insane in the asylums of this country. This assertion may sound strange, in view of the fact that writers on alienism, from Hippocrates and Aretæus to Esquirol and Charcot, have affirmed the genital origin of many cases of insanity in women, and in view of the fact that to-day every asylum report contains a list of gynecic conditions which are assigned as causes of insanity. The evidence supporting my statement comes, however, in part, from these very lists, the gross imperfections of which testify to the unfamiliarity of their authors with the present status of pathology as applied to the intra-pelvic viscera. It follows quite naturally that, if the conceptions of pathology are defective, the treatment which is based upon these conceptions must be equally faulty. But it is a matter of common information that the average alienist is either a mere brain doctor, or, worse still, a mere *geist* doctor, according to his physiologico-metaphysical preconceptions. In support of this statement, I have the word of that very erudite writer, Professor William B. Fletcher,[1] himself an accomplished alienist of the broader and more progressive stamp, to the effect that "the modern pathologist of mental disease shows a strong tendency to look for all kinds of alienation to changed structure of the white and gray matter of the brain, to softened or atrophied cells and fibers." The futility of this narrow-viewed investigation of insanity is frankly avowed by no less distinguished writers than Tuke and Sundby in the declaration that, "up to the present time, no definite and distinct lesion has been shown to accompany invariably any definite and distinct form of insanity." Certainly, in view of this state of affairs, the profession and humanity at large may with propriety protest, in the language employed by the talented Dr. Workman,[2] of Toronto, twenty-five years ago, that "the circular argument of starting with the

1. *Progress*, February, 1889.
2. *American Journal of Insanity*, 1864.

premises that, as the brain is the organ of the mind, and insanity is a disease of the mind, therefore the brain, in insanity, must be diseased, whether we detect the disease or not, has been too long deferred to."

In view of this statement of affairs by gentlemen connected with asylums, but who manifest a disposition to break the shackles of conservatism which bind their specialty, it would seem that, having failed, by means of freezing microtomes and immersion lenses, to demonstrate adequate central lesions in insanity, it is but proper to look for a peripheral origin of at least some mental disorders. Of all the cases promising a reward for such an inquiry are those of insanity in women. The highways over which morbific impulses may be transmitted from the frail organs of generation in women to the brain, through which at least intellectual aberrations must be manifested, are so abundant that one is surprised to learn of a scientific writer dissenting from the long-recognized doctrine of the genital origin of insanity in the female sex. We learn, however, that an author no less distinguished than Spitzka[1] declares, "that the grossest lesions of the female generative organs cannot alone cause insanity." This extraordinary declaration from a man who stands at the head of his department in America, and its apparent adoption in the practice of at least the vast majority of asylum physicians in this country, furnishes sufficient warrant for going into the question from the starting point of a few elementary facts.

NERVOUS RELATIONS OF THE FEMALE ORGANS OF GENERATION.

It would be presumptuous, in this presence, to go deeply into the nervous relations of the female organs of generation. It may not be amiss, however, to recall the fact that all of these organs have intimate connections with both the sympathetic and spinal systems of nerves, and that these systems of nerves in turn communicate with each other. Through these instrumentalities morbific impulses, originating in diseased conditions of the intra-pelvic viscera, may be telegraphed, as it were, either to

1. *Journal of Mental Sciences*, July, 1886.

those centers which preside over nutrition, or to those other centers which govern sensibility and motion. In the former instance, the sympathetic system furnishes the route of transit, and the result may be observed in the modified calibers of all the blood-vessels generally; in the perverted activity of all the glandular organs; in the unrythmical movements of all the hollow viscera and gland ducts; and in a more or less marked interference with ultimate tissue nutrition. In the latter instance, the cerebro-spinal system of nerves are involved, and the results are pain, and, may be, motor disturbances of either the voluntary or reflex character. In either case, the brain is reached, and we cannot fancy that impressionable organ receiving an impulse in excess of normal, without sustaining more or less functional disturbance, whether that disturbance be manifested by psychic phenomena or not. The perpetuation of this disturbing influence, through the persistence of the initial lesion in the distant viscera, is sufficient to convert the functional disturbance of the brain into an organic change, involving that organ. We can, therefore, appreciate the judicious utterance of Batty Tuke, when he says that—

"One great difficulty is to determine whether the 'cerebral' morbidities, which are apparent on microscopic examination, are of primary or secondary nature, whether they have been efficient causes of insanity, or whether they are merely the results of mal-nutrition of the brain."

And now, having indulged in these general reflections, let us pass from the abstract to the concrete.

### MENSTRUAL DISORDERS AND INSANITY.

The relationship of menstrual disorders to insanity may be either causal or consequent. Every gynecologist is familiar with the central nervous or cerebral origin of certain cases of amenorrhea, but I am not now discussing that class of cases. My business is with those cases in which the menstrual disturbance figures as the antecedent, and, logically, the causal condition. They are fairly illustrated by a case which I saw several years since in the practice of my friend, Professor Millikin, in which a robust young woman, in otherwise physically good health, had for some time complained of that family of symptoms which indicate disorder of the internal organs of generation. On one occasion, the diminishing menstrual flow failed to appear. Nervous symptoms supervened, and a few days later the unfortunate victim manifested the distressing phenomena of acute mania. A few days later, she was incarcerated in the Southern Ohio Asylum for the Insane, where she went from bad to worse, until death came to her relief after a series of months. Inquiry as to the treatment adopted in her case, brought the information that no attempt had ever been made to correct the almost manifest local mischief within the pelvis. Here was a case in which every circumstance pointed to a causal relationship between the gynecic state and the mania; and yet the fact was ignored, practically, if not absolutely, in the treatment adopted. I say practically, for the reason that the mere administration of iron and aloes, or similar medicaments, does not comprise a rational treatment of intra-pelvic disease. The course that should have been pursued in this case, when once it was beyond the sympathetic intermeddling of relatives, was to have placed her under anesthesia, subjected her to careful examination, and to skilful treatment based upon the revelations of that examination. I am aware that cases of this description can be managed, but with difficulty; that they are among the cases to which Skene, in his recent masterly treatise, alludes when he speaks of the obstacles to the successful local treatment of the insane; but, manifestly, the innocuous beneficence of anesthesia may be relied upon to at least assist in the solution of the problem. But before dismissing this case, I wish to state explicitly that the criticism of its treatment, in which I have indulged, must not be construed as a reflection upon the skill of the physicians at that time connected with the institution. I happen to know that they are gentlemen of liberal culture within their profession. The fault must lie at the door of the *system*, which appoints only three men to treat a half a thousand or more cases, and, in addition, attend to the various executive duties of the establishment. To treat one of these cases as I have suggested, would require a half an hour; and as there are generally at the same time from fifty to a hundred cases, not necessarily parallel, but yet equally demanding local treatment, it is obviously impossible to give them all the attention which they deserve.

PUBESCENT MANIA FROM DELAYED MENSTRUATION—RECOVERY.

In 1879, my friend Dr. Harris referred to me the case of a girl, eighteen years of age, with the following previous history: She had been a romping healthy girl up to her fourteenth year, when a marked change in her disposition was noted. She became nervous, introspective, and melancholy. The change was attributed to approaching puberty, but, as time went on, the flow failed to appear. In its stead, however, there occurred, each month, a marked exacerbation of all the neuritic phenomena, and each exacerbation was marked by an increasing severity, until finally, a year previous to the time she consulted me, they amounted to maniacal seizures of several days' duration. She had been under the care of a prominent specialist for nervous diseases in St. Louis, and had had treatment in two or more private sanitoria. In each instance, attention had been given to the state of her general health, with particular reference to her nervous system—treatment which all will admit was important, but which fell short because it was not directed at the initial, the causal lesion. On examination, I found an undeveloped uterus, and areas of great tenderness above the fornices of the vagina, indicative of ovarian hyperesthesia. She was put to bed, treated locally by the hot douche and galvanism, given a full diet and massage. Three weeks later we were rewarded by a slight show of menstruation and a modified maniacal seizure, and at the end of the next month we had a fairly well established flow and no mentral aberration whatever. The remaining hysteroid trouble gradually subsided under treatment during the next four months, at the end of which time she was dismissed cured. That was nearly ten years ago. Last week I received a letter from her, telling me she is the mother of two children, and that she has had no recurrence of either mental or nervous trouble since she passed from under my care. In this case there is great pertinence to the inquiry, Where was the essential lesion, central or peripheral? The sequel of the treatment stands as a sufficient reply.

MELANCHOLIA FROM PELVIC ABSCESS—OPERATION—CURE.

I recently reported to the Cincinnati Academy of Medicine, the case of a young lady who, in September of last year, applied to me for treatment. She was not physically robust and never had been, and her avocation, that of a teacher, had enfeebled her still more. Three years ago, she became conscious, by lumbar and sacral pains, supra-pubic discomfort, cystic irritability, leucorrhea, and painful menstruation, of the encroachments upon her health. Her nervous system yielded rapidly to these depressing influences until she entered one of the best equipped sanitoria in the State of New York, where for eight months she was treated by the most approved methods for neurasthenia. She came away but little better than when she went. Meantime her mental symptoms had grown worse, giving way as she did to a morbid subjectivity, until she could fairly "suck melancholy out of a song." The mental depression was most marked when she came to me; the indisposition to food was pronounced; neurotic emesis was annoying, and, on two separate occasions during the earlier stages of treatment, I thwarted her in serious attempts at suicide. On examination I found diffuse inflammatory infiltration of the pelvic cellular tissue, and, just above the right fornix of the vagina, the fluctuating point of an abscess was made out. After a little preparatory treatment, the requisite surgical measures were resorted to, with resulting recovery not only from the local mischief but from the mental disorder as well. In this case we may again ask, Was the lesion central or peripheral? In view of the manifest surgical indications in the case, indications which I endeavored to meet, and in view of the result, but one answer can be given. Was the case one of "toxic insanity?" or, Was it a "reflex insanity?" are interesting questions of differentiation which we have not time here to discuss. For our present purpose, the lesson which this case teaches is that local surgical interference was essential to its cure, and that as there are doubtless similar cases in the asylums that ought to be similarly treated, the asylums should be equipped with specialists to detect these conditions and to meet their indications.

### APHASIA FROM CERVICAL STENOSIS—OPERATION—RECOVERY.

The illustration of the peripheral origin of cerebro-psychic disturbance, to which I now invite your attention, is to my mind one of the most striking and conclusive. Mrs. D., aged 62, was referred to me by Dr. Mallory, of Hamilton, in February of this year. She was markedly aphasic, and had an offensive, muco-purulent discharge from the vagina; the latter, of course, being the condition which brought her to me. On examination, I found the case one of typical *vaginitis senilis*, resulting in occlusion of the upper segment of the canal, closure of the os uteri, and practical obliteration of the cervix. I at once put her under an anesthetic, tore adhesions within the vagina, and forcibly dilated the cervix. I should say that from two to three ounces of sanguino-purulent mucus escaped. The cavity was curetted, and then washed out with antiseptic solutions. The canal was kept patulous, and the irrigations were continued for a fortnight, when she was sent home cured. The interesting fact was that the aphasia began to subside from the moment the uterus was relieved of its contents, and that it had entirely disappeared when she went home. Her recovery remains complete. Here was a case in which, viewed entirely from the psycho-neurological standpoint, the lesion would have been located, according to Broca, in the third left frontal convolution; or, according to Meynert, in the island of Reil; but, viewed from the gynecological standpoint, the essential lesion, so far as the aphasia was concerned, actually existed in the sub-mucous ganglia, which, according to Frankenhäusen, comprise the terminii of the uterine nerves. It was here that the morbific impulse was originated, which, transmitted to the brain, induced functional perturbation of those centers directly involved in the production of the aphasic phenomena.

OVARIAN CYSTOMA AND INSANITY.

The influence of ovarian cystoma in the causation of insanity has not attracted much attention. From the fact that asylum reports and other literature of alienism are silent on this complication, whether causal or consequent of insanity, leads one to the conclusion that it does not exist. The next thought in the train thus started is that there must be something in the neurotic temperament to prevent the development of ovarian cysts, or else, considering the frequency of such tumors in women at large, there would certainly be an occasional case among the thousands of female inmates of our mad-houses. It has fallen to my lot to see one such case, a young woman under the care of Dr. Cook, of the Oxford Retreat, who consulted me with reference to an ovariotomy. The mental disturbance in this case had been coördinate in its development with the growth of the cyst. We agreed as to the expediency of an operation, but ill-advised friends interfered, and I lost sight of the case. Dr. R. Percy Smith, however, reports[1] a case in which the patient's insanity corresponded with the growth of the tumor. Ovariotomy was done, followed by prompt convalescence. There was improvement in the mental state from the start, and although there was not complete restoration of mental health, the hallucinations of smell were cured. In the discussion which followed, the statement was made that this was the first publicly recorded case of the kind in England. While my research has not been extensive, I have yet to encounter a similar record from American sources. If, indeed, as seems likely, unfortunate women are locked up in our bastile-like asylums, affected with not only insanity but the physical complication of an ovarian tumor, and are denied the beneficence of modern surgery, the offense is a most grievous one against humanity. In such a

1. *Journal of Mental Science*, July, 1886.

case, there is no sense in talking about cause or consequence; the only question to settle is: Will you, or will you not, give a crazy woman a chance for her life and for ultimate recovery?

HYSTERO-EPILEPTIC INSANITY FROM DISEASED UTERINE APPENDAGES—LAPARATOMY—CURE.

I am aware that the genital origin of hystero-epileptoid troubles, with or without psychical complications, and their cure by operative interference, has been a hotly contested question between alienists and gynecologists for a quarter of a century. I have, however, had some experience bearing so directly upon the point at issue, that I may be pardoned for briefly citing, at least, a single illustrative case. In 1884, with the assistance of Drs. Millikin and Skinner, I extirpated the appendages from a woman whom I had under observation for two years for hystero-epilepsy complicated with attacks of moliminal mania.[1] There were local symptoms in plenty to indicate organic mischief of the pelvic organs; but, in addition to this, her epileptic seizures were invariably preceded by a distinct ovarian aura. The operation was followed by complete cure of the nervous and mental disorders, and her health, in these particulars, remained perfect until her death, which occurred three years later from phthisis supervening upon pneumonia. The only case at all analogous to this that I have seen emanate from asylum circles, is one recorded by Dr. Strong, of the Cleveland asylum.[2] The patient had had recurrent attacks of mania, with increase of the trouble at the menstrual periods, during five years. Oöphorectomy was done by the late Dr. A. C. Miller, and the patient's mental trouble cleared up in five months. In both these cases—Dr. Strong's and my own—the changes in the ovaries were those of follicular degeneration. This is practically the same condition as that defined by Bucknill and Tuke, twenty years ago, as the most constant lesion of the ovary occurring in insane women. It is true that we do not hear of similar observations being made in

1. Transactions Ohio State Medical Society, 1886.
2. Annual Report N. O. Lunatic Asylum, 1887.

the *post-mortem* rooms of American asylums to-day, although it is a demonstrated fact that among women, taken as they come, the lesion is one of common occurrence. The fact may be accounted for by the statement of a man entirely familiar with asylum work in America,[1] to the effect that asylum physicians are not pathologists, but that "the duties of a superintendent of a large hospital for insane are rather executive than scientific." Would it not be well to fortify the "staff" of these institutions with pathologists who are competent to observe and record the conditions found? And then, too, rather than wait for these cases to die to find out what is the matter with them, would it not be well to have on the "staff" surgeons familiar with the diagnosis of intra-pelvic disorders, and skilled in the technique of abdominal operations? The primary mortality from these operations is small—vastly less than the ultimate death-rate in cases that are let alone but that really demand interference. It is high time for some experimental surgery to be done in this direction, even though healthy organs might occasionally be removed. I, for one, have no very great sentimental respect for either the ovaries or testicles of lunatics.

PUERPERAL INSANITY—LAPARATOMY—DRAINAGE—RECOVERY.[2]

I was called early in February of this year to see a case of puerperal insanity, five weeks after delivery. I found the patient with marked depression of all the mental faculties, and distinct hallucinations. The abdomen was distended and fluctuating. I made an incision, removed nearly three quarts of dark, offensive fluid, washed out the cavity, inserted a drainage-tube, and treated her by irrigation for three days, when the tube was taken out. The melancholia yielded somewhat, and the hallucinations subsided entirely. There was, however, in this case the potent intercurrent cause, heredity, which, with lactation, which was obstinately persisted in, doubtless accounts for the tardiness in the complete recovery from the melancholia.

1. Wm. B. Fletcher, M. D. *Progress*, February, 1889.
2. Academy of Medicine Proceedings. *Lancet-Clinic.*

### OTHER GENITAL REFLEXES IN INSANITY.

In 1858, Brown-Sequard pointed out the dependence of insanity, epilepsy, hysteria, and other affections, upon organic disease of the female generative organs. Baker Brown put these teachings into practice, and furnished clinical proofs of their correctness. He, however, fell a martyr to the professional bigotry which his doctrines excited. Now, however, that the rancor of that day has died out, his book on "The Curability of Certain Forms of Insanity, Epilepsy, Catalepsy, and Hysteria in Women" can be read with interest, and, modified by the later revelations of science, his teachings can be followed with safety. These subjects, however, together with the mental disorders of the menopause, the perversions of the sexual instinct, nymphomania, etc., must be passed. I can only pause to mention the fact that Flechsig has reported a case of recovery from hysterical insanity immediately following the removal of a uterine fibroid.[1] And here I must drop the pathological phase of this question.

The considerations which I have presented justify me, I think, in the following

### CONCLUSIONS.

1. There is a causal relationship existing in many cases between disease of the female generative organs and insanity in women.

2. The rational treatment of these cases consists in (1) the removal of the causal lesion; and (2) in the treatment of the consequent functional or organic disturbance.

3. The adoption of these principles of practice involves the appointment of gynecologists to the staffs of asylums.

### ASYLUM REFORMS.

It would now seem that my task is at an end, but such, in fairness to myself, is not the case. The question of the peripheral origin of insanity is much broader than the gynecic

1. *Neurologisches Centralblatt*, 19–20, 1885.

problem which I have been discussing. Dr. Loomis, of New York, has called the attention of the profession to the frequent origin of insanity in optical defects. Diseases of the ear as causes of insanity has long been recognized. Fletcher, in the article from which I have already quoted, affirms the origin of insanity in disease of various abdominal and other organs. Ought not such patients to have the benefit of treatment from an opthalmologist, an aurist, and a specialist in internal medicine? And then, too, the insane are liable to all the acute diseases, including surgical conditions. In fact, the mortality lists show that the majority of all the deaths in asylums are from acute diseases. In the name of humanity, then, ought not these unfortunates to have the benefit of the best special skill in all the departments of practice? This brings me to my final suggestion: Abolish the superintendent and assistant system, and, instead, appoint a staff made up of representative specialists, supplemented by an adequate corps of house physicians. And here let me drop the subject, which, I feel, is far less exhausted than is my audience.

311 Elm Street.

